# The Regulatory Microenvironment in Feathers of Chickens Infected with Very Virulent Marek’s Disease Virus

**DOI:** 10.3390/v14010112

**Published:** 2022-01-09

**Authors:** Jegarubee Bavananthasivam, Nadiyah Alqazlan, Mohammadali Alizadeh, Ayumi Matsuyama-Kato, Jake Astill, Raveendra R. Kulkarni, Shayan Sharif

**Affiliations:** 1 Department of Pathobiology, Ontario Veterinary College, University of Guelph, Guelph, ON N1G 2W1, Canada; jkrubee@gmail.com (J.B.); nadiyah.alqazlan@gmail.com (N.A.); alizadem@uoguelph.ca (M.A.); matsuyam@uoguelph.ca (A.M.-K.); jake.s.astill@gmail.com (J.A.); 2 Department of Population Health and Pathobiology, College of Veterinary Medicine, North Carolina State University, Raleigh, NC 27607, USA; ravi_kulkarni@ncsu.edu

**Keywords:** Marek’s disease virus, feather, T regulatory cell, chicken, MD vaccine

## Abstract

Vaccines against Marek’s disease can protect chickens against clinical disease; however, infected chickens continue to propagate the Marek’s disease virus (MDV) in feather follicles and can shed the virus into the environment. Therefore, the present study investigated if MDV could induce an immunoregulatory microenvironment in feathers of chickens and whether vaccines can overcome the immune evasive mechanisms of MDV. The results showed an abundance of CD4^+^CD25^+^ and CD4^+^ transforming growth factor-beta (TGF-β)^+^ T regulatory cells in the feathers of MDV-infected chickens at 21 days post-infection. In contrast, vaccinated chickens had a lower number of regulatory T cells. Furthermore, the expression of TGF-β and programmed cell death receptor (PD)-1 increased considerably in the feathers of Marek’s disease virus-infected chickens. The results of the present study raise the possibility of an immunoregulatory environment in the feather pulp of MDV-infected chickens, which may in turn favor replication of infectious MDV in this tissue. Exploring the evasive strategies employed by MDV will facilitate the development of control measures to prevent viral replication and transmission.

## 1. Introduction

Marek’s disease (MD) in chickens is caused by an oncogenic herpesvirus, named Marek’s disease virus (MDV) [1]. MDV is shed from feathers of infected chickens and is transmitted via the respiratory tract of chickens through inhalation of contaminated dust or feather follicle dander [2,3]. After spreading throughout the body, MDV produces enveloped infectious viruses in the feather follicle epithelium (FFE), which are shed along with feathers and feather dander [4]. Although MD vaccines control clinical disease, vaccine-induced host responses are unable to prevent viral replication in FFE and transmission of MDV to the environment [5,6,7]. Moreover, the viral load in feather follicles is higher than that in the spleen of MDV-infected chickens [6]. These apparent differences in viral numbers between lymphoid tissues and the productive replication site suggest that immune responses to MDV in feather follicles may not be effective, hence, favoring the successful replication of the virus for transmission. Importantly, Marek’s disease vaccines, including herpesvirus of turkeys (HVT) and CVI988, can replicate and induce host immune responses in feathers [7,8,9]. However, until now, little research has been carried out to investigate how the microenvironment in chicken feathers supports virus replication and whether MD vaccines can overcome such an environment. Here, we consider the immunoregulatory microenvironment as a biological site consisting of inhibitory or regulatory cells and cytokines that suppress host immune responses against productive replication of MDV in feathers. Therefore, the current study was carried out to examine whether immunoregulatory responses are induced in feathers in favor of MDV replication and if such regulatory responses can be overcome by vaccine administration.

## 2. Materials and Methods

### 2.1. Chickens

One-day-old specific pathogen-free white leghorn chicks were kept in the animal isolation facility at the Ontario Veterinary College, University of Guelph. The Animal Care Committee of the University of Guelph approved all experimental procedures carried out in this study in accordance with the guidelines of the Canadian Council on Animal Care.

### 2.2. Vaccine and Virus

The HVT FC-126 vaccine strain was obtained from Boehringer Ingelheim Animal Health Canada Inc as a generous gift. The CVI988 vaccine was purchased from Merck Animal Health (Intervet Canada Corp, Kirkland, QC, Canada). Chickens were challenged with a very virulent strain of MDV (RB1B), which had been propagated in chickens [10].

### 2.3. Experimental Design

One-hundred and sixty-eight chickens were randomly assigned to 6 experimental groups. Chickens in each group were housed separately in Horsfall units. The manufacturer’s recommended dose (0.2 mL per chicken) of HVT or CVI988 vaccines were administered subcutaneously to chickens at two weeks of age. For collecting sufficient cells from the pulp of growing feathers, chickens were vaccinated at two weeks of age and subsequently infected with MDV. Phosphate-buffered saline (PBS) was used as a control. PBS containing 500 plaque-forming units of RB1B was injected into the abdomen of each chicken at 18 days of age. The various experimental groups were as follows: MDV-challenged group (MDV, *n* = 30), HVT-vaccinated, and MDV-challenged group (HVT+MDV, *n* = 30), CVI988-vaccinated and MDV-challenged group (CVI988+MDV, *n* = 30), HVT-vaccinated group (HVT, *n* = 27), CVI988-vaccinated group (CVI988, *n* = 27) and a PBS group (PBS, *n* = 24) as a negative control. From each chicken, feathers and spleen were collected at 4-, 10- and 21 days post-infection (dpi) to assess RB1B MDV genome levels, cellular composition and expression of immune system genes.

### 2.4. DNA and RNA Extraction and Real-Time Polymerase Chain Reaction (PCR)

To evaluate RB1B MDV genome levels in the feathers and spleen, genomic DNA was extracted from these tissues [11]. A total of 100 ng of DNA was used with primers amplifying the *Meq* gene of RB1B MDV in real-time PCR with the SYBR green master mix. To measure RB1B genome levels in the challenged group, RB1B meq primers were used. Although the CVI988 vaccine strain also contains meq, it contains two isoforms that differ by a 178bp insertion. These are not present in RB1B MDV [12,13].

As described previously, feather tips were used to extract RNA using TRIzol (Life Technologies, Burlington, ON, Canada), and 1 μg of DNase-treated RNA was used to synthesize cDNA [14]. The synthesized cDNA was used to evaluate the expression of immunoregulatory genes by real-time PCR. The primers used in this study, Table 1, were purchased from Sigma-Aldrich Canada (Oakville, ON, Canada).

### 2.5. Isolation of Cells from Feathers and Spleen

Mononuclear cells were prepared from feathers as described previously [19]. Briefly, the entire pulp was collected from feather tips in 0.5 mL of 0.1% collagenase-dispase solution (Collagenase type IV, Life Technologies, Carlsbad, CA, USA; Dispase II, Sigma-Aldrich, Oakville, ON, Canada). The pulp cell suspension was subsequently incubated at 40 °C for 15 min and strained through a 40 μm strainer with ice-cold PBS. The collected cells were washed with ice-cold PBS twice at 250× *g* at 4 °C, and the final cell pellet was collected in fluorescent activated cell sorting (FACS) buffer (PBS containing 1% bovine serum albumin).

Spleen cell suspensions were prepared by crushing spleens. Following rinsing with Hank’s balanced salt solution, cells were filtered through a 40-μm nylon cell strainer and re-suspended in complete RPMI medium (Invitrogen, Burlington, ON, Canada) supplemented with 10% fetal bovine serum (Millipore-sigma, Oakville, ON, Canada) and 1% Penicillin-Streptomycin (Gibco, Carlsbad, CA, USA). To obtain mononuclear cells, cell suspensions were overlayed on 4 mL of Histopaque-1077 (Sigma, Oakville, ON, Canada) followed by centrifugation at 400× *g* for 20 min. Cells at the interface were harvested in a complete RPMI medium and washed twice.

### 2.6. Flow Cytometry Analysis

MOXI Z cell counter (Orflo, Ketchum, ID, USA) was used to count the isolated cells from spleen and feathers. Hundred µL of each cell suspension at the concentration of 5 × 10^6^ cells/mL in a complete RPMI medium were plated in round bottom 96 well plates. Subsequently, fluorescent monoclonal antibodies were added to the washed cells and kept in the dark for 30 min at 4 °C for staining the cells. The surface staining antibodies used in this study were PB conjugated mouse anti-chicken CD3, PE-Cy7 conjugated mouse anti-chicken CD4, PE conjugated mouse anti-chicken CD8, FITC conjugated human anti-chicken CD25, and APC conjugated mouse anti-chicken TGFβ. CD25-FITC was obtained from Bio-Rad (Mississauga, ON, Canada), and the rest of the antibodies were purchased from SouthernBiotech (Birmingham, AL, USA). Fixable Live/Dead near-IR fluorescent reactive dye (Thermo Fisher Scientific, Waltham, MA, USA) was used to stain dead cells for removing them from subsequent analyses. After surface staining of cells, they were fixed in 2% paraformaldehyde following two times wash with FACS buffer.

Using a FACScanto II flow-cytometer (BD Bioscience, San Jose, CA, USA) 200,000 events per feather and 100,000 events per spleen were collected. FlowJo Software v.10 (Tree Stat, Ashland, OR, USA) was used to analyze the data. The gating strategy was performed as follows (Supplementary Figure S1): initial gating was carried out to exclude cell debris in the SSC -A vs. FSC-A plot. Then, at the SSC-A vs. live/dead plot dead cells were excluded, and live cells were selected. Next, singlets were chosen in FSC-W vs. FSC-H plot and SSC-W vs. SSC-H plot. Then, SSC-A vs. CD3 plot was gated to select all T cells. Subsequent T cell subsets were gated using CD4, CD8, CD25, and TGF-β markers from this gating. Subsequently, cell counts were determined based on the frequency of the cells.

### 2.7. Statistical Analysis

Advanced relative quantification software in the Light-Cycler 480 II system was used to determine the relative expression of target genes to the housekeeping gene, β-actin. Logarithmically transformed gene expression data and flow cytometry data were analyzed by two-way ANOVA and Tukey’s multiple comparison test. MDV genome levels were analyzed by a non-parametric statistical method, Kruskal–Wallis test. Geometric mean of relative expression ± standard error of the mean was used to plot the gene expression data in the graph. If *p*-value was ≤0.05, the results were considered significant.

## 3. Results and Discussion

**Figure 1:**
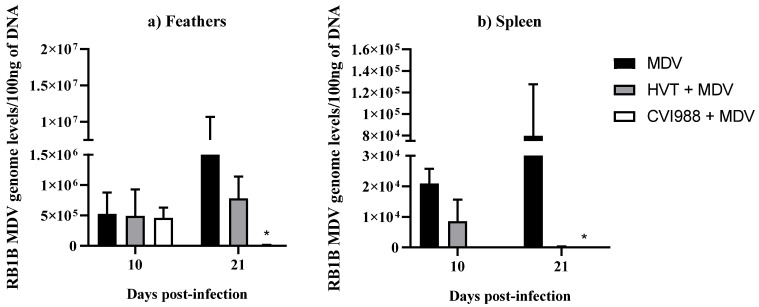
RB1B MDV genome levels in feathers and spleen. RB1B MDV genome levels per 100 ng of DNA were calculated from (**a**) feathers (*n* = 8) and (**b**) spleen (*n* = 8) collected at 10 and 21 dpi. Viral load data were analyzed by Kruskal–Wallis test. If *p*-value was ≤0.05, it was considered statistically significant (*). Each group was compared with the MDV-challenged group. Error bars indicate the standard error of the mean.

MDV genome levels were determined by extracting DNA from the feathers and spleen in MDV-challenged chickens followed by real-time PCR (Figure 1). As expected, the viral load was significantly decreased in both feathers and spleen in the MDV-challenged group that received the CVI988 vaccine compared with MDV-challenged chickens (Figure 1a,b). This reduction in MDV load coincides with a previous observation where a significant difference in viral load was reported at 14 dpi in feathers following in ovo vaccination with CVI988 and MDV infection [20]. Although MDV genome copy numbers were reduced in HVT-vaccinated and MDV-challenged chickens compared with the MDV-challenged group, it was not statistically significant, which agrees with our previous study [9]. Further, regardless of vaccination, viral load was several-fold higher in feathers compared with the spleen tissue of chickens (Figure 1) [20,21]. In agreement with this study, a higher MDV genome load has been found in feathers compared with the spleen following MD vaccination [6]. These findings indicate the presence of a favorable environment for MDV replication in feathers.

**Figure 2:**
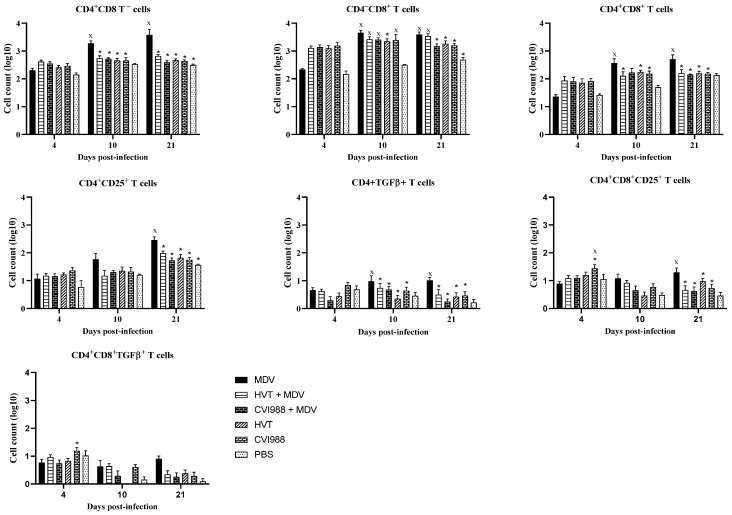
Evaluation of subsets of T cells in feathers (*n* = 6) at 4, 10, and 21 dpi. Cells were isolated from the feather pulp from feather tips, and analyzed by flow cytometry at various time points following the MDV challenge. Two-way ANOVA and Tukey’s multiple comparison test were used to analyze these data. If *p*-value was ≤0.05, it was considered statistically significant when compared with PBS (x) or MDV (*). Cell counts on the *Y*-axis are presented on a logarithmic scale. Error bars indicate mean ± standard error.

The cells collected from feather pulp and spleen were analyzed by flow cytometry for the presence of T cell subsets, including CD4^+^CD25^+^ and CD4^+^ TGFβ^+^ T regulatory cells. These regulatory cells are essential for controlling the exaggerated immune responses and maintaining immune homeostasis [22]. A gradual increase in CD4^+^ and CD4^+^CD8^+^ T cells was observed in the feathers of MDV-challenged chickens (Figure 2), which agrees with previous findings [23]. On the other hand, a reduction in CD4^+^ and CD4^+^CD8^+^ T cells was observed in feathers of vaccinated and MDV-challenged chickens compared with MDV infected chickens at 10 and 21 dpi (Figure 2). Similar to our findings, Islam et al. also reported slight depletion of T cells in HVT-vaccinated chickens than MDV received chickens [24]. In contrast, a reduction in CD4^+^ cells in the spleen and blood was described following vaccination with CVI988 compared with unvaccinated chickens at the early stage of immune responses [25].

**Figure 3:**
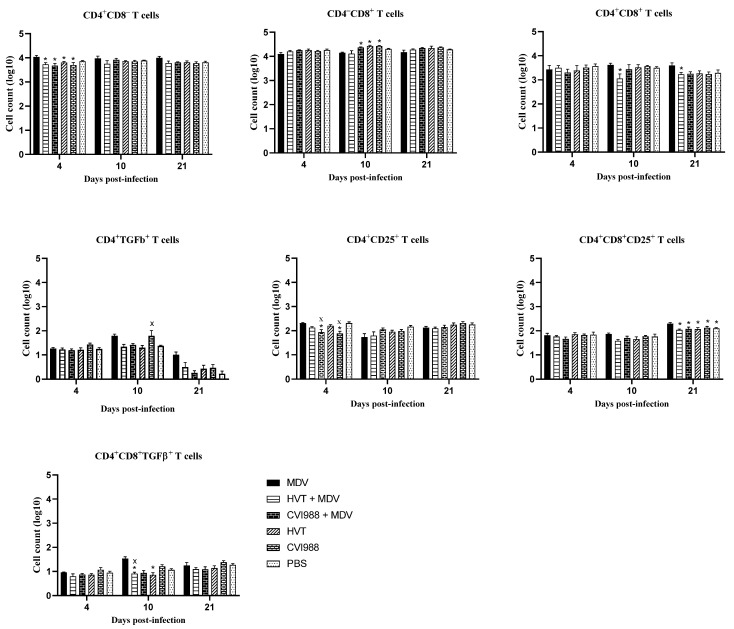
Evaluation of T cell subsets in the spleen (*n* = 6) at 4, 10, and 21 dpi. Spleen cells were analyzed by flow cytometry at different time points following the MDV challenge. Two-way ANOVA and Tukey’s multiple comparison test were used to analyze these data. If *p*-value was ≤0.05, it was considered statistically significant when compared with PBS (x) or MDV (*). Cell counts on the *Y*-axis are presented on a logarithmic scale. Error bars indicate mean ± standard error of mean.

In chickens, CD25 and TGF-β containing CD4^+^ T regulatory cells exist in various immune system tissues [26,27]. Here, we demonstrated the detection of these T regulatory cells in the feathers of MDV-challenged chickens. In vaccinated and challenged chickens, CD4^+^ CD25^+^ and CD4^+^ TGF-β^+^ cells were decreased in feathers at 10 and 21 dpi (Figure 2). Spleen T regulatory cells were also assessed to compare them with feathers (Figure 3). No difference in spleen T regulatory cell numbers was observed between vaccinated chickens and MDV-challenged chickens. T regulatory cells modulate T helper type 1 and cytotoxic T cell immune responses required for protection against viral infections [22]. Therefore, the existence of T regulatory cells in the feathers of MDV-challenged chickens may correspond with the inhibition of T cell function and replication of infectious MDV in feathers. Involvement of TGF-β expressing T regulatory cells in the pathogenesis of MDV infection has already been demonstrated [26]. Our findings on the increased number of TGF-β^+^ T regulatory cells and MDV load in feathers agree with this report.

The expression of programmed cell death (PD)-1 receptor, PD ligand (PDL)-2, interleukin (IL)-10, TGF-β, and cytotoxic T lymphocyte-associated antigen (CTLA)-4, is necessary for the inhibitory function of T regulatory cells [28]. Among these markers, CTLA-4 and PD-1, which are members of the CD28 superfamily immunoreceptors, counteract activation and function of effector T cells. PD-1 binds with PDL-1 and PDL-2 on CD4^+^ and CD8^+^ T cells and suppresses the secretion of pro-inflammatory cytokines [29]. In addition, T regulatory cells control immune responses by secreting anti-inflammatory cytokines, IL-10 and TGF-β [30]. Perforin is important for the function of cytotoxic T cells for the elimination of virus-infected cells and tumor cells in the host [31]. Therefore, the expression of perforin was assessed in this study to evaluate the activity of cytotoxic T cells in feathers where the enhanced regulatory function is expected.

**Figure 4:**
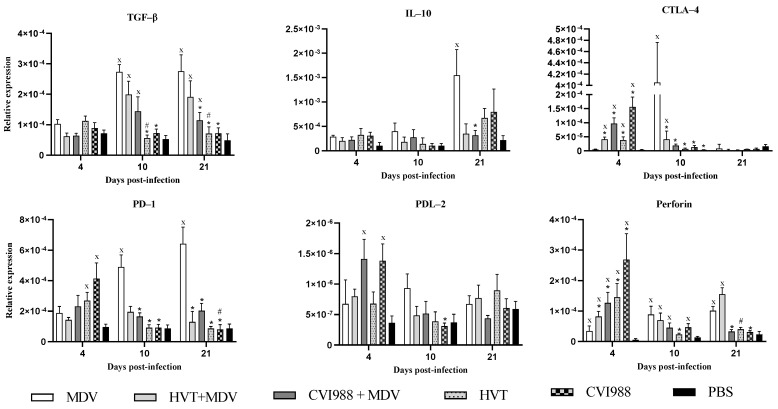
Relative expression of the immunoregulatory genes in feathers. Gene expression of TGF-β, IL-10, CTLA-4, PD-1, PDL-2, and perforin in feathers was determined based on the expression of β-actin at 4, 10, and 21 dpi. Logarithmically transformed data were analyzed by two-way ANOVA and Tukey’s post-test. *Y*-axis indicates the geometric mean of relative expression ± standard error of the mean. If *p-* value was ≤0.05, it was considered statistically significant when compared with PBS (x) or MDV (*) or HVT+MDV vs. HVT (#) or CVI988+MDV vs. CVI988 (#).

The expression of TGF-β, PD-1, PDL-2, IL-10, CTLA-4, and perforin was evaluated as markers of immunoregulatory functions in the feather. At 10 and 21 dpi, TGF-β and PD-1 expression significantly declined in vaccinated chickens compared with MDV-challenged chickens (Figure 4). TGF-β can prevent naïve T cell activation and proliferation. TGF-β can also induce apoptosis in activated T cells to dampen cytotoxic function, which favors replication of the virus [32].

The finding of increased expression of PD-1 in feathers of MDV-challenged chickens compared with PBS control chickens at 10 and 21 dpi coincides with a similar pattern of expression reported in splenocytes and CD4^+^ T cells in the spleen in previous studies [18,33]. These observations suggest that CD4^+^ T cells possibly contributed to the expression of PD-1 in feathers. The progressive upregulation of PD-1 corresponds to the increased CD4^+,^ and CD4^+^CD8^+^ T cell counts in MDV-challenged chickens, which may indicate the exhaustion of these T cells. The occurrence of these exhausted cells may be one of the mechanisms employed by MDV for the evasion of host responses. In addition, CTLA-4 expression was also decreased at 10 dpi in vaccinated chickens (Figure 4), which shows the reduction in inhibitory effect on effector T cells. Importantly, CTLA-4 has been shown to be increased in feathers and spleen CD4^+^ T cells of MDV-challenged chickens [18], which point to the possible involvement of CTLA-4 in the pathogenesis of MDV. Based on the observation that CTLA-4 is downregulated in feathers, it may be concluded that vaccines counteract the MDV effect for inducing an immunoregulatory microenvironment.

In contrast to the expression of regulatory genes, perforin expression was initially high in vaccinated chickens at 4 dpi and then subsided in feathers with no difference among the various groups (Figure 4). Perforin is crucial for the cytotoxic activity of T cells to control viral infection. The increased expression of perforin in the feathers of vaccinated chickens indicates the cytotoxic activity of effector cells, possibly involving the elimination of MDV-infected cells and interrupting the regulatory milieu in feathers. A high expression of perforin might have caused a reduction in MDV numbers in this tissue in vaccinated chickens. However, the expression of perforin at the initial time point might have been overcome by the presence of regulatory factors, such as TGF-β and T regulatory cells in feathers. It is also possible that due to the exhaustion of effector cells, perforin expression declined at the later stages of pathogenesis. Therefore, it may be concluded that MD vaccination interrupts the regulatory microenvironment in feathers. This possibly leads to a reduction in MDV replication, thereby reducing shedding and transmission of infectious MDV to the environment and chickens. However, this remains to be formally investigated.

Overall, our findings provide evidence for increased CD4^+^CD25^+^ and CD4^+^TGF-β^+^ T regulatory cell numbers in feathers of MDV-challenged chickens. However, these cells were significantly reduced when chickens were immunized with HVT or CVI988 vaccines before the MDV challenge. In addition, TGF-β and PD-1 transcripts were high in feathers of MDV-challenged chickens. These observations indicate the occurrence of an immunoregulatory microenvironment in feathers of MDV-infected chickens. This microenvironment may support the productive replication of MDV in FFE and subsequent transmission to unvaccinated chickens. Furthermore, age-related resistance might have influenced MDV infection in this study. Our current study expands the understanding of the biological properties in the feathers of chickens during MDV infection and MD vaccination which will assist in understanding the evasive mechanism employed by MDV. Identifying additional molecular factors that enable replication of infectious MDV in feathers and studying the immune evasive mechanisms utilized by the virus are necessary for developing intervention approaches to prevent replication and transmission of MDV.

## Figures and Tables

**Table 1:** Target genes, primer sequences and references used for real-time PCR.

Genes	Primer Sequences 5′-3′	References
β-actin	F: CAACACAGTGCTGTCTGGTGGTA R: ATCGTACTCCTGCTTGCTGATCC	[15]
IL-10	F: AGCAGATCAAGGAGACGTTC R: ATCAGCAGGTACTCCTCGAT	[6]
TGF-β	F: CGGCCGAGATGAGTGGCTC R: CGGGGCCCATCTCACAGGGA	[16]
Perforin	F: ATGGCGCAGGTGACAGTGA R: TGGCCTGCACCGGTAATTC	[17]
CTLA-4	F: CAAGATGGAGCGGATGTACC R: TGGCTGAGATGATGATGCTG	[18]
PD-1	F: GTGATTGTGCTGCTGCTCTTTG R: GAACTCCAGCACACCGTAGTC	[18]
PDL-2	F: CTTCACATTACCAGCGTCAGG R: GACTGGCATATAAGAGCAAAC	[18]
meq	F: GTCCCCCCTCGATCTTTCTC R: CGTCTGCTTCCTGCGTCTTC	[11]

## Data Availability

The datasets generated and analyzed during the current study are available from the corresponding author on reasonable request.

## Supplementary Materials

The following supporting information can be downloaded at: https://www.mdpi.com/article/10.3390/v14010112/s1, Figure S1: The gating strategy with the representative FACS plots.
