# Supplementary material for: The Regulatory Microenvironment in Feathers of Chickens Infected with Very Virulent Marek’s Disease Virus
Source: Viruses. 2022 Jan 9;14(1):112. doi: 10.3390/v14010112 (PMC8781056; doi:10.3390/v14010112)

The Regulatory Microenvironment in Feathers of Chickens Infected with Very virulent Marek's Disease Virus

Jegarubee Bavananthasivam [1,†], Nadiyah Alqazlan [1], Mohammadali Alizadeh [1], Ayumi Matsuyama-Kato [1], Jake Astill [1,‡], Raveendra R. Kulkarni [2] and Shayan Sharif [1,*]

Supplementary figure S1: The gating strategy with the representative FACS plots.

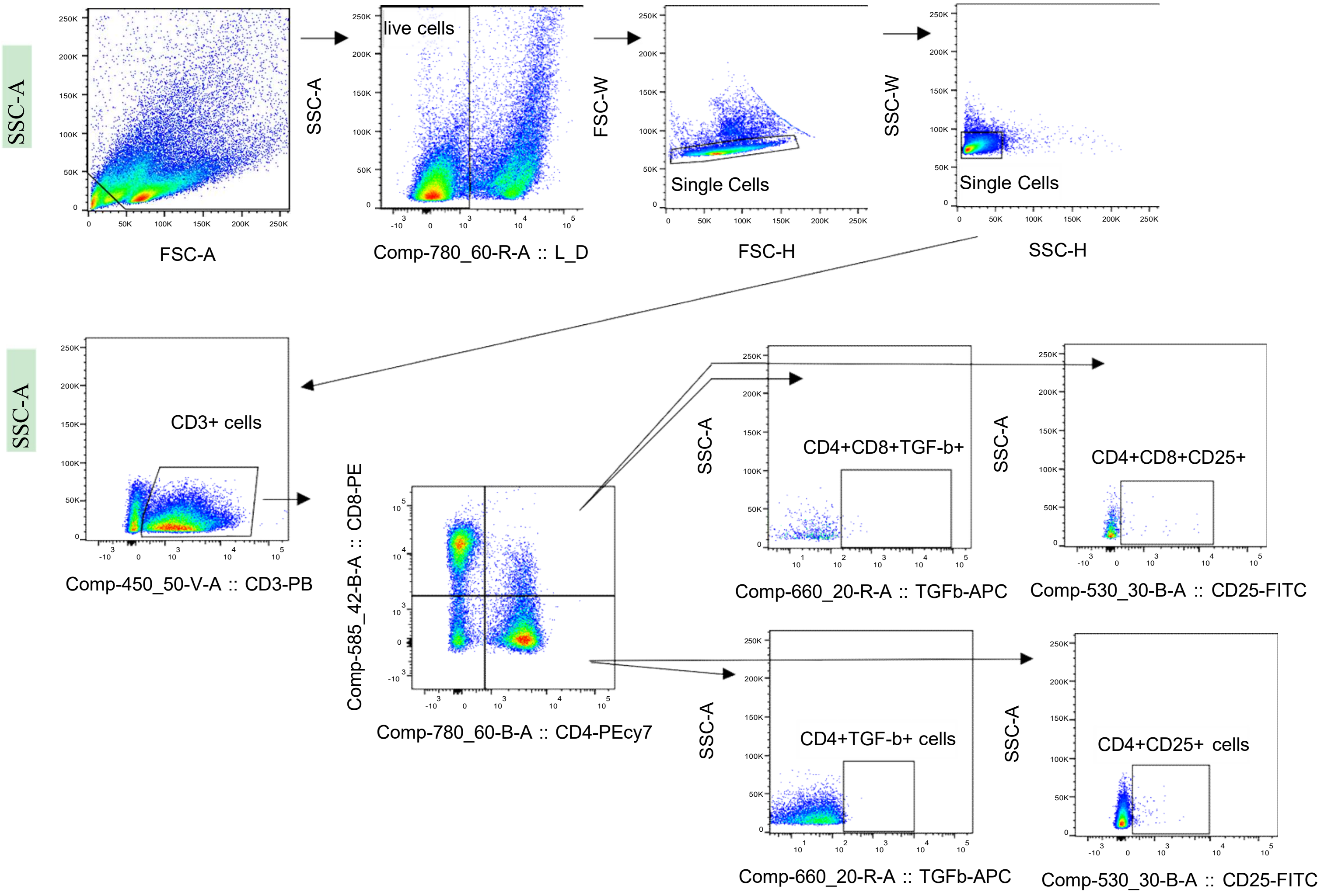

Supplement: Supplementary file 1 [file viruses-14-00112-s001.zip › viruses-1527323-supplementary.pdf]
